# Indiana State Dental Association

**Published:** 1866-08

**Authors:** Benj. D. House


### Proceedings of Societies.

### INDIANA STATE DENTAL ASSOCIATION.

REPORTED BY BENJ. D. HOUSE.

The above organization met, pursuant to adjournment, at the rooms of the Young Men's Christian Association at Indianapolis, Ind., Tuesday, June 26th, 1866, at two o'clock, P. M.

Present—Drs. C. C. Burgess, E. M. Morrison, J. B. Harlan, A. E. Purcell, J. Knapp, W. R. Webster, A. T. Keightly, M. Wells, G. A. Wells, and J. F. Johnston.

In the absence of the President, the meeting was called to order by Vice-President Burgess.

Dr. Morrison, Chairman of the Executive Committee, submitted the following report:

### ORDER OF BUSINESS.

1st, Reports of committees; 2nd, Admission of members; 3d, Election of officers; 4th, Retiring President's address; 5th, Installation of officers; 6th, Reading of original essays; 7th, Discussion of the following topics in order:

1st, Dental materia medica, including anæsthetics; 2nd, Exposed pulp, alveolar abscess, sensitive dentive; 3d, Extraction of teeth; 4th, Gold filling; 5th, Tin and other cheap fillings; 6th, Dental fees and ethics; 7th, Vulcanized rubber and other artificial appliances generally; 8th, Dental progress and new inventions; 9th, Miscellaneous business.

On motion the report was accepted and the committee discharged.

On motion, the following Committee on Membership was appointed by the Chair to report at once: Drs. Johnson, Wells, and Keightley.

The committee reported the following names as candidates for admission to the Association:

Drs. H. H. Morrison, Greencastle; S. M. Cummins, Elkhart;

J. K. Jameson, Shelbyville; J. D. Robertson, Dublin; W. L. Heiskill, Indianapolis; J. M. Ball, Centreville; W. H. Gilbert, Peru; N. H. Allen, Anderson; W. F. Morrill, New Albany; M. Strong, Indianapolis; E. Totten, Crawfordsville; M. H. Chappell, Knightstown; T. H. Martin, Lebanon; G. W. Cast, Thornton; Wm. Glenn, Muncie.

On motion of Dr. Knapp the rule was suspended and a vote taken *vive voce*.

The candidates were unanimously elected.

On motion, Dr. Pierce, of Philadelphia, was elected an honorary member of the Association.

The Secretary stating that it was absolutely necessary for him to be absent for the evening, Dr. Gilbert was appointed Secretary, *pro tem*.

The following officers were elected to serve for the ensuing year: President, Dr. Knapp, of Fort Wayne; Vice-Presidents, Dr. Webster, of Richmond; Dr. Morrill, of New Albany; Dr. A. E Purcell, of Indianapolis; Secretary, Dr. J. F. Johnston, of Indianapolis; Treasurer, Dr. G. A. Wells, of Indianapolis.

On motion, the Association adjourned until 8 o'clock, P.M.

### First Day—Evening Session.

The Association met promptly at 8 o'clock.

The meeting was called to order by the President.

Report of the afternoon session read and approved, after which the following names were presented and accepted for membership: Dr. J. S. Snoddy, of Lafayette, and Dr. J. E. Swallow, of Indianapolis.

On motion, the regular order of business was taken up.

The essayists appointed to read essays before the Society not being prepared, Dr. Merritt Wells was continued as such until the next annual meeting.

An amendment was made that Dr. Wells should present his essay on the following morning.

Dr. Moore, of Lafayette, then read an essay on "Alveolar Abscess", in which the subject under consideration was ably canvassed, and various remedies for this disease were mentioned according to the ideas and experience of the essayist.

On motion, the thanks of the Association were tendered to Dr. Moore for his able essay.

Dr. Webster, of Richmond, then raised questions in regard to certain opinions given by Dr. Moore.

After which a general discussion of the disease under consideration took place, in which various members of the Association participated. The question was discussed to considerable extent, and various members of the Association gave a synopsis of their treatment of the disease. Dr. Morrison, of Noblesville, read a statement of a case that came under his care, and his manner of treatment and the remedies used which effected a radical cure; after which several members expressed opinions in regard to various questions raised by the reading of the statement of Dr. Morrison.

On motion of Dr. Morrill the Association adjourned until 9 o'clock the following morning.

### Second Day—Morning Session.

The Association met, pursuant to adjournment, and was called to order by the President.

The Secretary then read report of the previous day's proceedings.

On motion, the report was accepted.

The regular order of business was then taken up: 1st, Examination of candidates for admission to the Association.

On motion of Dr. Webster, Dr. Moore was requested to furnish a copy of the essay read the previous evening for publication.

On motion Dr. Forbes, of St. Louis, who was in attendance, was elected an honorary member of the Association.

The unfinished business of the previous evening was then taken up—the consideration of the alveolar abscess.

A general discussion then took place, in which several members participated. Dr. Chappell then presented several specimens of bone for examination extracted in an operation on a case of the disease under discussion. Causes and effects of said disease were then quite ably and generally discussed. The case reported by Dr. Chappell was canvassed to a considerable extent, in which various different opinions were expressed by members of the Association.

Dr. Keightly then stated a case similar to that reported by Dr. Chappell, manner of treatment, &c., which elicited further discussion.

Dr. Wells then stated a case at present under his care, in which the inferior incisors were absent and a portion of the bone seemed detached, being movable and presenting a porous and blackened appearance. The movable piece wedge-shaped, apex downwards. Asked advice in regard to its removal. The gum at certain points seemed firmly attached at the lower portion of the loosened bone. Inquired in regard to the proper treatment. Was able to present the case in a very clear manner by the use of a casting of the case under consideration.

Dr. Johnston responded, stating that from the concise manner in which the case was presented that it was nearly as easy to diagnosis the case as though the patient were present. In his opinion it was a clear case of necrosis. His treatment of a case of the kind described would be the removal of the diseased portions of the bone by the use of bone forceps and chisels, or whatever instrument most convenient for the accomplishment of the desired object. Considered it absolutely essential that every portion of the diseased bone should be removed, if possible. Where the periosteum was firmly attached it would be sufficient evidence of the part in question being in a healthy condition, hence this should be made the line of demarkation.

The Doctor recommended such constitutional treatment as the patient seemed to require—the use of tonics, &c. The question was raised, was it properly in the province of a dentist to prescribe such remedies.

The Doctor admitted that there was a difficulty. Still, it might be done if the dentist was in good standing in his profession. By consulting with the family physician his advice as a general thing would be taken. In the absence of a physician the dentist might with perfect propriety prescribe in the premises.

Dr. Webster coincided with Dr. Johnston with the exception of treating the patient with tonics. Did not think it in the province of a dentist to do so.

Dr. Johnston differed from him in all cases where the family physician could not be consulted, and where he was sure of the proper remedies.

The Chair then made some remarks in regard to the subject under discussion, and called for an expression of opinion from Dr. Forbes, of St. Louis, who responded in remarks of a clear, concise nature, coinciding and endorsing Dr. Johnston's manner of treatment, stating his experience in several different cases and his manner of treatment.

Dr. Forbes has the faculty of saying what he wishes and making himself understood in a manner not to be mistaken.

The Chair then called on Dr. Forbes to give his manner of treatment of alveolar abscess and filling of cavities.

Dr. Forbes responded, and entered at some length into his own experience and manner of treatment of the disease under consideration. Gave a statement of the Association Meeting at St. Louis. Remarked that few liked to state their failures, yet as much was to be learned from failures as from successes.* He, being the oldest member of the profession at the St. Louis Convention, told of his failures, and other members followed suit. One man made a statement which proved him one of the most consummate scoundrels extant. If there were any scoundrels present the Doctor wanted them to tell

*Can not agree with this statement.—[Ed.

of it. (The man was only a scoundrel in not understanding his profession.)

The remarks made by Dr. Forbes elicited questions and remarks from Dr. Johnston and others, which were answered by the Doctor, some of them in a manner that created considerable mirth.

Dr. Johnston was then called upon and gave his experience and manner of treatment of the disease under discussion.

General remarks were elicited by the statements of Dr. Johnston, in which several members of the Association participated. The manner of Dr. Johnston's treatment was quite generally endorsed.

On motion the further discussion of the subject of alveolar abscess was discontinued, and the next subject in order taken up.

Dr. Wells was called upon to furnish an essay as per resolution of last evening upon the subject of Anæsthetics. Not being prepared with a written statement, he gave a verbal account of his understanding of the subject. His remarks were plain, and gave evidence that the speaker had studied the subject, although not prepared with a written essay.

Dr. Wells asked for the experience of any member who had used nitrous oxide.

Dr. Keightley responded, and gave a description of its effects from personal experience, giving quite a facetious description, which caused some visible smiling.

Dr. Knapp then stated a case treated in this manner that recently came under his notice.

A general discussion then took place.

Dr. Burgess made some remarks in regard to the use of chloroform. Thinks it has been too much decried. Personally prefers it to nitrous oxide. Asked how many deaths from the use of chloroform had come under the notice of the Association during the past year? No response. Stated the symptoms of the patient under the influence of chloroform. Didn't think it desirable to give patient a sufficient quantity to make him perfectly insensible. A general discussion followed.

Dr. Johnston made remarks upon the subject at some length.

Question asked Dr. Johnston.—Do you administer stimulants after giving chloroform?

Advised its not being given until *after* the use of chloroform. The chloroform in itself acts as a stimulant at first, and the patient being naturally depressed after its influence passes off, needs toddy then if at all.

Dr. Morrill enquired of Dr. Forbes if it was not the usual practice in St. Louis to administer "toddy" previous to administering chloroform.

The Doctor answered that he was ready to give that mode a personal practical test on the spot. After which he spoke at some length; stated his experience and mentioned some very interesting cases.

Dr. Forbes thinks it better to use "toddy" after the chloroform is administered, if at all.

On motion, the meeting adjourned until 2 o'clock, P. M.

### Second Day—Afternoon Session.

The meeting was called to order by the President at 2 o'clock, P. M.

On motion, Dr. Purcell was invited to state his experience in the use of nitrous oxide gas. He did so at some length. Uses it almost entirely.

The question of adjournment was then called up by Dr. Moore, and a motion was made by him that the Association adjourn after the night session that P. M.—Carried.

On motion, topic of extracting teeth was ommitted, and the next in order taken up—gold filling.

Dr. Forbes was called upon to speak upon this subject but declined, and Dr. Moore opened the discussion. Had rather listen to persons of more experience than to speak himself. Has been in practice fourteen years. Thinks he has made great advances. Uses adhesive and sponge gold. Carefully clears the cavities. Commences the filling by introducing adhesive gold into the pit by the use of the mallet, gradually building up. Having teeth well separated. If he finds any deficiency, builds on them. Used soft foil two years ago; uses adhesive foil altogether now. Only objection length of time required to perform the operation.

Question by the Chair—Should time be considered, if the results justified.

Answer—Not if the patient was able to pay for that time.

Dr. Morrill then took the floor and stated his opinions. Believes in making thorough excavation of all cavities. Used a diamond-pointed drill; uses Dunlevy's gold foil No. 3; commences the bottom with anchorages of gold foil. In large cavities thinks four or five anchorages should be made.

Dr. Johnston was requested to take the floor. Advocates use of non-adhesive foil for filling crown cavities in molars and condenses with mallet as he goes. Thinks that one method only should not be entirely depended upon. Thinks as a general thing the non-adhesive gold condensed with mallet makes more perfect work than with adhesive filling, in a large class of cavities. Thinks building out can be done only with adhesive gold; thinks, however, that in most cases building out is not desirable, being too attractive and not sufficiently lasting.

Dr. Forbes then took the floor and explained his system by diagram. Asked Dr. Johnston to explain manner of filling a given cavity.

The Doctor gave his opinion; was questioned in regard to the depth of a cavity required in order to fill with cylinder. In filling an inferior molar would take a wedge of hard wood, drive between, separating the teeth and pressing back the gums, then uses paper and napkin for the purpose of absorbing the saliva and closing the ducts. If a superficial cavity would fill with adhesive gold. Explained his system with a tooth, which elicited many questions, and were answered by diagramatical explanation.

The President then explained his method. Uses adhesive gold—nearly all crystal gold foil condensed with mallet. Has but one mode, to excavate carefully and fill up as above decribed.

Dr. Forbes then explained his system by diagram, which cannot be reported unless by drawing. When he cannot see the whole cavity, breaks away the enamel until it can be seen. Makes the walls nearly upright; uses a cone drill; always cleans off the outside of the teeth at the start. Has cylinders made of all sizes; prepares them when he has nothing else to do; places the cylinder in the cavity and then "sends it home." Uses but one kind of gold; the price of gold not as much value as time; is sure that the cavity thus far is solidly filled before placing in the last cylinder.

Drs. Forbes and Johnston's methods very nearly similar. Desultory questions followed.

Dr. Forbes thinks that sufficient gold is seldom used in the filling of cavities.

The President made a few remarks, stating that he doubted not good plugs could be made in many different ways. Mentioned filling made by him nine years ago—still good; would not be pleased with the work to-day, but the teeth have been preserved.

Dr. Moore was asked how he dried the plug when by mischance it became wet. New serrates the gold, &c.; always has two assistants. Stated that he did not fully understand Dr. Forbes' explanation.

Dr. Forbes took the floor, and in response to a remark made by the Chair that each one was peculiarly attached to his own method, stated that with him it was not so, as he adopted all that seemed to him improvements on his own method.

Dr. Webster was called on. Had no fixed method; "just went right on and filled them." Has fillings which have been in twenty years; uses his gold in strips introduced by the wedge process; uses cylinders occasionally; serrated instruments, small pointed; sometimes uses much force in large cavities.

Dr. Morrill asked some one to describe the manner of filling bicuspids.

Dr. Totten being called upon by the Chair, stated his manner of filling a cavity in the above mentioned teeth. Uses a silver wedge; forces it down between the teeth; introduces his gold from the grinding surfaces, filling from the bottom and condensing with mallet, making the wedge a wall of the tooth for the time being; uses the drill to sink pits. Thinks iron, if rightly shaped, would answer the purpose as well as the wedge above mentioned.

Dr. Morrill thinks when by accident the cavity becomes wet while under the process of filling that it should be washed out with ether and new pits drilled.

On motion, discussion was discontinued in regard to gold filling, and tin and other cheap fillings taken up.

Dr. Cast then gave his experience with Woods' metallic alloy. Had not used it very extensively.

Dr. Morrison had used it but little; it worked well; considers it nearly equal to tin; worked easily, &c.

Dr. Burgess told of a case where tin fillings had stood nineteen years in the under molars on the grinding surface the upper molars being present.

Dr. Martin stated a case of the second inferior molar on grinding surface being filled with tin, which stood twenty-one years and kept the teeth in a good state of preservation.

Dr. Robertson stated his experience. Had not used Woods' alloy to any great extent. Told of a case where tin filling had remained forty years in good condition on a grinding surface. Thinks tin the best of all cheap fillings.

Dr. Keightley endorsed tin fillings.

Dr. Burgess stated the case of Rev. Mr. Green, who had an upper tooth filled with old-fashioned silver amalgam, when the nerve was destroyed one day and filled the next.

Dr. Forbes stated that if the same pains were taken any tooth could be filled with tin that could be filled with gold, with the exception of building up. Thinks tin the best non-conductor that can be used; use in the same manner that gold was once used in ribbons; advised the non-usage of Woods' alloy; advised Lawrence's amalgam when necessary to use such an article; encloses the amalgum in a piece of buckskin and subjects it to a vice power, after which he subjects it to heat; advises the use of tin for the purpose of learning to manipulate gold; will never use amalgam in a bicuspid or any of the front teeth; in filling one of the latter named teeth he breaks out the intervening space between the cusps, whether sound or not; "if there is no hole he makes one."

On motion of Dr. Johnston the further discussion was discontinued, and miscellaneous business taken up.

Dr. Johnston read an invitation to attend the Third Annual Meeting of the Central States Dental Association, to be held at Louisville, Ky., commencing July 17th, 1866.

On motion, the invitation was received and cordially accepted.

On motion, delegates were elected to attend the American Dental Association, to be held at Boston; Mass., to commence on the last Tuesday in July.

The following were elected: Dr. Richardson, of Terre Haute; Dr. Morrill, of New Albany; Dr.s Hunt and Johnston, of Indianapolis; Dr. Knapp, of Fort Wayne; Dr. Webster, of Richmond; Dr. Moore, of Lafayette; Dr. Keightly, of Greencastle, and Dr. Jamison, of Shelbyville.

On motion, the Secretary was empowered to fill vacancies that might occur.

The thanks of the Association were tendered to Dr. Forbes for his attendance.

On motion, $8 00 were voted for the use of the room.

On motion, it was ordered that the next annual meeting be held at Indianapolis on the last Tuesday of June, 1867.

On motion, the meeting adjourned until 8 o'clock; P. M.

### Second Day—Evening Session.

Meeting called to order by Vice-President Morrill.

On motion, reading of minutes was suspended.

On motion, the afternoon discussion was resumed.

Dr. Morrill made some remarks in regard to cheap filling. Finds in his experience with Woods' filling that oxide gathers under the filling after it has been in place some time; thinks it has been quite generally ignored.

Dr. Burgess stated that he never used it in but two cases; sometimes uses plastic alloy for the time being until the permanent teeth are ready.

Dr. Cummings used it for about six months, became disgusted with it and discontinued to use, only for temporary use.

Dr. Wells never used it to fill a tooth. Stated that a lady melted it in hot water in a case where it had been used to fasten on a plate tooth.

Dr. Morrison, of Greencastle, never used it for filling. Sometimes used it as a cement.

Dr. Tauton never uses it as a filling. In several cases where he has removed it found that the teeth have decayed under the filling. The decay seemed of a yellow cast; thinks it produces a soreness at the time of usage; generally uses amalgam; thinks tin the best; uses amalgam because it is easier manipulated.

Dr. Pifer had not used it to any great extent. Discovered the same discoloration mentioned by the foregoing speaker; thinks Woods' office licence; not on the square; prefers amalgam.

Dr. Moore. Never used it to any extent; don't like the idea of cheap fillings; a tooth worth saving is worth saving well; prefers amalgam; takes the same care with the latter as with gold; makes little difference in price between amalgam and gold; sometimes where the walls of the cavity are thin uses os artificial as a support. In his experience was not satisfied with Woods' alloy. In filling with gold thinks the dentist should educate his patients in regard to care of the teeth; if decayed teeth are owing to ill condition of blood patient should be treated for the same; thinks it for the pecuniary interest of dentists to do so; has several families under his treatment; don't think decayed teeth need to exist to any great extent if the people had proper instruction and treatment at the hands of the profession. Thinks the condition of the blood regulates the perfection of all dental operations.*

